# Country tobacco laws and article 11 of the WHO Framework Convention on Tobacco Control: a review of tobacco packaging and labeling regulations of 25 countries

**DOI:** 10.1186/1617-9625-11-23

**Published:** 2013-11-06

**Authors:** Ayodeji J Awopegba, Joanna E Cohen

**Affiliations:** 1 Institute for Global Tobacco Control, Johns Hopkins Bloomberg School of Public Health, Baltimore, MD, USA

**Keywords:** FCTC, Tobacco control, Tobacco packaging and labeling, Health warnings, Compliance

## Abstract

**Background:**

Urgent, evidence-based tobacco control efforts have been advocated by the WHO through the Framework Convention on Tobacco Control (FCTC) articles and guidelines. The level of implementation of these guidelines varies by country and region. This paper identifies areas of alignment and non-alignment of country tobacco laws with respect to the FCTC’s article 11 requirements, which lists guidelines for regulating tobacco packaging and labeling.

**Methods:**

Countries from each of the six WHO regions were ranked by number of smokers and 25 countries were selected, representing countries from all WHO regions with the highest number of smokers. A scoring guide based on the FCTC article 11 requirements was created and used to rank country tobacco laws and assess levels of alignment as well as identify common areas of weakness and strength.

**Results:**

Across the countries examined, laws were generally strong in mandating the display of health warning messages on the front and back of cigarette packs and cartons. However, they were deficient in prohibiting the display of emission yields, and placing warnings at the top of the principal display area, as well as requiring health messages on tobacco’s negative social and economic outcomes.

**Conclusion:**

Country tobacco packaging and labeling laws can be strengthened by greater compliance with the FCTC article 11 guidelines.

## Background

Tobacco use remains the leading, single most preventable cause of death globally; the current annual death rate attributable to tobacco use stands at about 5.4 million deaths per year and is projected to increase to more than 8 million deaths annually by 2030 if urgent tobacco control efforts are not instituted [1]. The Framework Convention on Tobacco Control (FCTC), created to respond to the looming tobacco epidemic, as well as protect and promote global public health, articulates provisions that aim to reduce the supply and demand of tobacco globally. Adopted in November 2008, Article 11 guidelines [2] lists provisions for the regulation of tobacco product packaging and labeling.

Tobacco companies are increasingly using the cigarette package as a primary marketing vehicle, as is evident from this statement from the industry: “*Our final communication vehicle with our smoker is the pack itself. In the absence of any other marketing messages, our packaging…is the sole communicator of our essence*” [3]. The significant advertising potential of the cigarette packet is underscored by the persistent push back of the tobacco industry against plain packaging and other measures to reduce tobacco use [4].

Strong health warning messages can influence the decision to initiate or quit smoking [5,6], and these measures can be implemented at virtually no cost to government [7]. In addition, there is strong public support for strong health warnings, even among smokers [8-12]. However, it is not clear the extent to which countries are enacting strong tobacco packaging regulations that are consistent with the FCTC article 11 guidelines. This paper assesses the level of compliance of country tobacco laws with the mandatory components of the FCTC article 11 guidelines, and identifies common areas of weakness in tobacco labeling laws in the countries that contribute the most to the global burden from smoking across all six WHO regions.

## Methods

### Country selection

Countries with the highest numbers of smokers in each WHO region were selected for this study. Absolute number of smokers for each country was estimated from age- and sex-standardized adult daily smoking prevalence for the year 2009 [7] and country total population for 2010 [13]. Countries in each WHO region were ranked, from highest to lowest, by estimated number of smokers. The first six countries in the European Region, as well as the first five countries in each of other WHO regions were selected to give a sample with the highest contribution to the global burden of smoking across all WHO regions. In instances where country laws were not available, or where verified translations were not accessible electronically, the next country on the list was selected, provided the numbers of smokers in both countries were comparable. In the African and Eastern Mediterranean regions, where these numbers were far apart, fewer countries were selected. This led to a final selection of 25 countries: six countries in the European region, five countries in the Americas, South-East Asia and Western Pacific regions, and two countries in the African and Eastern Mediterranean regions.

The countries by region are as follows: Africa (South Africa, Kenya); The Americas (Mexico, Canada, Brazil, Argentina, USA); South-East Asia (Nepal, Thailand, India, Bangladesh, Indonesia); Europe (Spain, Turkey, Poland, United Kingdom, Ukraine, Russia); Eastern Mediterranean (Pakistan, Egypt) and Western Pacific (Australia, Malaysia, Philippines, Vietnam, China).

### Scoring criteria

We examined the FCTC article guidelines and distinguished required guidelines from optional recommendations by careful examination of how they were worded. Required guidelines were considered those that used words such as “must”, “should”, or “shall”; while optional guidelines were classified as those that used words such as “may” or “can”, or contained phrases like “Parties should consider…”.

The resulting scoring criteria contained 19 mandatory health warning components grouped under the following five categories: location, size, message content, language and display of misleading descriptors. We also assessed optional recommendations such as the use of pictograms, contrast, and the provision of a “quit line” number.

We used the scoring criteria thus created to assess each country’s compliance with FCTC article 11 guidelines on tobacco packaging and labeling. We extracted country tobacco laws from the Campaign for Tobacco-Free Kids website http://www.tobaccocontrollaws.org[14], as this was considered a reliable source of verified translations of the tobacco packaging and labeling laws of different countries. We awarded one point for meeting each required guideline and one-half point where guidelines partially complied with the FCTC requirements. If a country’s laws did not precisely reflect what the FCTC guidelines specify, no point was awarded. Thus, higher total scores indicate greater alignment of the laws with the guidelines.

### Analysis

Scores across all article 11 requirements were totaled for each country to reflect the overall level of alignment with the guidelines. Within WHO Regions, countries were ranked from highest to lowest total score.

## Results

Out of a maximum of 19 points, total scores ranged from 18 (Australia) to 4 (Indonesia). Three countries in the selection (USA, Argentina and Indonesia) have not ratified the FCTC. Across all countries examined, laws were generally strong in requiring that health warning messages are displayed on the front and back of cigarette packs and cartons. However, they were generally weak in prohibiting the display of emission yields, and placing warnings at the top of the principal display area (which is, in most cases, the front and back, or the widest part of the package), as well as requiring health messages on tobacco’s negative social and economic outcomes.

### Results by category

#### Location

Most countries (n = 23) in the selection required warnings on both packs and cartons, except Russia and Indonesia, that did not require health warnings on cartons (Table 1). Less than half of the countries in the selection (n = 11) required that warnings are placed at the top of the principal display area (PDA). Brazil, Indonesia, Philippines and India required warnings to be placed on only one PDA. Kenya, Egypt, Indonesia, China, Vietnam did not mandate that health warnings be placed at the top of the PDA, or placed where they would not be damaged by opening the pack, or that they are positioned where they would not be obstructed by mandatory markings on the packs. In this selection, Mexico, Spain, Turkey Nepal and Australia were the most compliant with regard to the requirements on location, scoring the maximum points for this category, while Indonesia ranked least.

**Table 1:** Characteristics of country laws, with respect to location of health warnings on cigarette packs

*Country	**Ratified FCTC**	**Year of ratification**	**Warning on pack and carton**	**Front and back**	**Top of PDA**	**Opening does not damage warning**	**Not obstructed by tax stamps, etc.**	**Total**
*Africa*								
South Africa	YES	2005	1	1	1	1	0	4
Kenya	YES	2004	1	1	0	0	0	2
*Americas*								
Mexico	YES	2004	1	1	1	1	1	5
Canada	YES	2004	1	1	0	1	1	4
Brazil	YES	2005	1	1	0	0	1	3
Argentina	NO		1	1	0	0	1	3
USA	NO		1	1	1	0	0	3
*Eastern Mediterranean*								
Egypt	YES	2005	1	1	0	0	0	2
Pakistan	YES	2004	1	1	1	0	0	3
*Europe*								
Spain	YES	2005	1	1	1	1	1	5
Turkey	YES	2004	1	1	1	1	1	5
Poland	YES	2006	1	1	0	1	1	4
UK	YES	2004	1	1	0	1	1	4
Ukraine	YES	2006	1	1	0	1	1	4
Russia	YES	2008	0.5	1	0	1	0	2.5
*South-East Asia*								
Nepal	YES	2006	1	1	1	1	1	5
Thailand	YES	2004	1	1	1	0	0	3
India	YES	2004	1	1	0	1	1	4
Bangladesh	YES	2004	1	1	1	0	0	3
Indonesia	NO		0.5	0.5	0	0	0	1
*Western Pacific*								
Australia	YES	2004	1	1	1	1	1	5
Malaysia	YES	2005	1	1	1	0	1	4
Philippines	YES	2005	1	1	0	1	1	4
VietNam	YES	2004	1	1	0	0	0	2
China	YES	2005	1	1	0	0	0	2

*Countries are ranked within each of the six WHO regions by overall level of compliance with FCTC article 11 guidelines.

#### Size

Most countries were generally compliant with the requirements on size. South Africa and Indonesia were the only countries in this analysis whose health warnings were not required to cover at least 30% of the principal display area (PDA) (Table 2).

**Table 2:** Characteristics of country laws, with respect to size of health warnings on cigarette packs

*Country	**Not less than 30% of PDAs**	**Bold, legible text**	**Total**
*Africa*			
South Africa	0	1	1
Kenya	1	1	2
*Americas*			
Mexico	1	1	2
Canada	1	1	2
Brazil	1	1	2
Argentina	1	1	2
USA	1	1	2
*Eastern Mediterranean*			
Egypt	1	1	2
Pakistan	1	1	2
*Europe*			
Spain	1	1	2
Turkey	1	0	1
Poland	1	1	2
UK	1	1	2
Ukraine	1	0	1
Russia	1	0	1
*South-East Asia*			
Nepal	1	1	2
Thailand	1	1	2
India	1	1	2
Bangladesh	1	1	2
Indonesia	0	1	1
*Western Pacific*			
Australia	1	1	2
Malaysia	1	1	2
Philippines	1	1	2
VietNam	1	1	2
China	1	0	1

*Countries are ranked within each of the six WHO regions by overall level of compliance with FCTC article 11 guidelines.

#### Misleading descriptors

Countries generally aligned poorly with the FCTC guidelines by not prohibiting the display of emission yields, and by failing to require the display of relevant qualitative emissions like Benzene. Though Brazil, Egypt, Malaysia and China ban the display of misleading descriptors, they do not prohibit the stealthy use of colors, and other insignia that could give a false impression that one brand is safer than another (Table 3). Mexico and Australia were the most compliant, getting all points under the category of prohibiting all forms of misleading descriptors on packs, whereas country tobacco laws from the USA, Pakistan, Russia, Bangladesh, Indonesia and the Philippines did not prohibit misleading descriptors, in any form, on packs and scored no points in this section.

**Table 3:** Characteristics of country laws, with respect to prohibition of misleading descriptors on cigarette packs

*Country	**Prohibition of term, descriptor, trademark or figurative or other sign that may be deceptive**	**Prohibition of display of emission yields**	**Display of relevant qualitative emissions such as benzene**	**Total**
*Africa*				
South Africa	1	0	0	1
Kenya	0	1	1	2
*Americas*				
Mexico	1	1	1	3
Canada	1	0	1	2
Brazil	0.5	0	1	1.5
Argentina	1	0	0	1
USA	0	0	0	0
*Eastern Mediterranean*				
Egypt	0.5	0	0	0.5
Pakistan	0	0	0	0
*Europe*				
Spain	1	0	0	1
Turkey	1	0	1	2
Poland	1	0	1	2
UK	1	0	0	1
Ukraine	1	0	0	1
Russia	0	0	0	0
*South-East Asia*				
Nepal	1	0	1	2
Thailand	1	0	1	2
India	1	0	0	1
Bangladesh	0	0	0	0
Indonesia	0	0	0	0
*Western Pacific*				
Australia	1	1	1	3
Malaysia	0.5	0	1	1.5
Philippines	0	0	0	0
VietNam	1	0	0	1
China	0.5	0	0	0.5

*Countries are ranked within each of the six WHO regions by overall level of compliance with FCTC article 11 guidelines.

#### Rotation

In this selection, only Indonesia and Bangladesh did not require health warnings to be rotated. Though rotation of health warnings was required by the rest, about half of country tobacco laws (n = 14) were still vague on the frequency of rotation, or the range of packs that the rotation sequence must apply to (Table 4).

**Table 4:** Characteristics of country laws, with respect to rotation of health warnings

*Country	**Rotation required**	**Rotation format specified**	**Range of packs to which rotation is applicable is specified**	**Total**
*Africa*				
South Africa	1	1	1	3
Kenya	1	1	1	3
*Americas*				
Mexico	1	1	1	3
Canada	1	1	1	3
Brazil	1	1	0	2
Argentina	1	1	1	3
USA	1	0	0	1
*Eastern Mediterranean*				
Egypt	1	1	0	2
Pakistan	1	1	1	3
*Europe*				
Spain	1	0	1	2
Turkey	1	1	1	3
Poland	1	1	1	3
UK	1	1	1	3
Ukraine	1	0	0	1
Russia	1	1	0	2
*South-East Asia*				
Nepal	1	1	1	3
Thailand	1	0	0	1
India	1	1	0	2
Bangladesh	0	0	0	0
Indonesia	0	0	0	0
*Western Pacific*				
Australia	1	1	1	3
Malaysia	1	0	1	2
Philippines	1	1	0	2
VietNam	1	1	0	2
China	1	0	0	1

*Countries are ranked within each of the six WHO regions by overall level of compliance with FCTC article 11 guidelines.

#### Message content

In this selection, only Spain required health warnings that covered all five components of the requirements under the category “Message content”. Most countries (n = 22), except Spain, Ukraine and Egypt, did not require health warnings about the adverse economic and social outcomes related to smoking on their packs. All countries in this analysis required warnings that talked about the adverse health effects of smoking (Table 5).

**Table 5:** Characteristics of country laws, with respect to message content of health warnings on cigarette packs

*Country	**Advice on health effects**	**Cessation**	**Addictive nature**	**Adverse economic and social outcomes**	**Impact on family and friends**	**Total**
*Africa*						
South Africa	1	1	1	0	1	4
Kenya	1	0	0	0	1	2
*Americas*						
Mexico	1	0	1	0	1	3
Canada	1	1	1	0	1	4
Brazil	1	1	1	0	1	4
Argentina	1	1	1	0	0	3
USA	1	1	1	0	1	4
*Eastern Mediterranean*						
Egypt	1	0	1	1	1	4
Pakistan	1	0	0	0	0	1
*Europe*						
Spain	1	1	1	1	1	5
Turkey	1	1	1	0	1	4
Poland	1	1	1	0	1	4
UK	1	1	1	0	1	4
Ukraine	1	0	1	1	1	4
Russia	1	1	1	0	1	4
*South-East Asia*						
Nepal	1	0	0	0	0	1
Thailand	1	0	0	0	1	2
India	1	0	0	0	0	1
Bangladesh	1	0	0	0	1	2
Indonesia	1	0	0	0	0	1
*Western Pacific*						
Australia	1	1	1	0	1	4
Malaysia	1	0	0	0	0	1
Philippines	1	0	0	0	1	2
VietNam	1	0	0	0	0	1
China	1	0	0	0	0	1

*Countries are ranked within each of the six WHO regions by overall level of compliance with FCTC article 11 guidelines.

#### Language

All countries’ laws under review required that health warnings be printed in at least one of the principal language of the country, in alignment with the FCTC guidelines on article 11.

#### Optional recommendations

In this selection, only South Africa, Mexico, Canada, Brazil, Argentina, Spain, Poland, the United Kingdom, Thailand, Australia and Malaysia provided a quit line number on their packs (Table 6). South Africa, Kenya, Poland, Indonesia, Philippines and China did not require graphic pictograms. Indonesia, China, Turkey and Ukraine did not explicitly state that warnings should use contrasting colors for the background of the text.

**Table 6:** Characteristics of country laws, with respect to optional health warning components of the FCTC

*Country	**Quit line**	**Pictograms**	**Contrast**	**Total**
*Africa*				
South Africa	1	0	1	2
Kenya	0	0	1	1
*Americas*				
Mexico	1	1	1	3
Canada	1	1	1	3
Brazil	1	1	1	3
Argentina	1	1	1	3
USA	1	1	1	3
*Eastern Mediterranean*				
Egypt	0	1	1	2
Pakistan	0	1	1	2
*Europe*				
Spain	1	1	1	3
Turkey	0	1	0	1
Poland	1	0	1	2
UK	1	1	1	3
Ukraine	0	1	0	1
Russia	0	1	1	2
*South-East Asia*				
Nepal	0	1	1	2
Thailand	1	1	1	3
India	0	1	1	2
Bangladesh	0	1	1	2
Indonesia	0	0	0	0
*Western Pacific*				
Australia	1	1	1	3
Malaysia	1	1	1	3
Philippines	0	0	1	1
VietNam	0	1	1	2
China	0	0	0	0

*Countries are ranked within each of the six WHO regions by overall level of compliance with FCTC article 11 guidelines.

## Discussion

This cross-country study of tobacco packaging and labeling laws showed that even countries that have ratified the FCTC are yet to align their laws to the highest standards of the FCTC article 11, especially with regard to the diversity of the content of health warnings, location of health warnings on the PDA of packs, and prohibition of misleading descriptors on cigarette packs.

It is important that health warning messages continue to reflect the extensiveness of the effects tobacco use can have on its users and those around them. Tobacco companies have historically obfuscated the facts about the addictive nature of nicotine, as well as the far-reaching adverse effects of smoking on health and the environment [15]. Consequently, many smokers, including non-smokers, have underestimated the extreme addictive nature of nicotine and the impact of their smoking habit on their health and those around them [16,17]. A combination of warnings that cover issues on health effects of smoking with adverse social and economic outcomes, addictive nature of nicotine, cessation and the impact of smoking on family and friends, as required by the FCTC, can be more powerful in convincing individuals who differ in what motivates them to initiate or quit smoking.

This study also detects a consistent weakness with respect to location of health warnings. Many countries do not require that health warnings be placed at the top of the principal display area. In addition, many country laws do not require health warnings to be located where they would not be obstructed by required markings on packs, or damaged/concealed with the opening and closing of packs. Most countries in the selection (except Mexico, Spain, Turkey, Nepal and Australia) do not meet all the requirements for location of health warning labels as required by the FCTC. Though large warnings have been shown to be effective by both smokers and non-smokers [18,19], placing them at the top of the PDA can further enhance their effectiveness and noticeability.

Most country laws in the selection did not prohibit the use of all forms of misleading descriptors on packs, except Australia and Mexico, which comply with all the requirements of the FCTC with respect to this category. Countries' laws were especially weak in prohibiting the display of quantitative emission yields on their packs. Users of these products may still ascribe lower risks to brands that have lower levels of tar, carbon monoxide or nicotine, attenuating the effect that the prohibition of the use of misleading terms such as “mild” “light”, may have had.

Six countries in the selection (South Africa, Kenya, Poland, Indonesia, Philippines and China) are yet to mandate the use of health warnings that contain pictograms. It is also important to note that most of these are low-and middle-income countries, where health literacy may be relatively low. Though the use of pictograms is not a requirement, countries can strengthen the impact of their warning labels by using graphic color images. Strong warnings that utilize graphic pictograms, and not just text, are shown to be more effective in getting the attention of users, conveying the significance of the text warning and ultimately inducing a change in the perception of risk by the users [18,20-27]. Studies have shown that smokers tend to notice health warnings with pictures more than they do warnings without [21,28]. Pictograms would convey a stronger message, especially in low-literacy settings, or in cases where text warnings are very weak in conveying the harms of tobacco use.

Strong health warning messages can influence the decision to initiate or quit smoking [5,6], and these measures can be implemented at no cost to governments [7]. Some countries like Canada [19,29,30], Australia [11], Brazil [31], Singapore [32] and Thailand [33] have seen significant change in perceptions and attitudes toward smoking following implementation of some of these FCTC-recommended best-practices in health warning display. Barriers to implementing best practices in tobacco packaging and labeling, as stipulated by the FCTC, would vary by country. Countries should share their successes and challenges, and collaborate on possible strategies to strengthen their tobacco laws. Sustained efforts to enact, mandate and enforce the evidence-driven guidelines outlined by the FCTC will enable countries to protect the health of their citizens and reduce mortality and illness from tobacco.

### Strengths and limitations

This study examines tobacco packaging and labeling legislation in countries that contribute the most numbers of smokers to the global burden from smoking across all six WHO regions. However, these findings are subject to at least four limitations. First, unofficial translations of country tobacco laws [14] were used to assess compliance with the FCTC provisions. Due to limitations of translation, certain wordings or expressions may not be accurately represented. However, these translations were carefully verified by in-country lawyers and experts, as well Campaign for Tobacco-Free Kids staff in Washington DC, and give a clear understanding of country tobacco laws. Second, this study examines tobacco regulations as written, not as practiced. Some countries may actually meet the FCTC requirements in practice, even though their laws do not. For example, Canada’s health warnings are placed at the top of the PDA, even though this is not specified in the legislation. Conversely, some countries may have laws that are compliant with the FCTC requirements, but are not enforced. Some examples include Vietnam and the US, whose new laws have not yet come into full effect. Third, this study examines laws that pertain only to manufactured cigarettes. Fourth, the unavailability of verified translations of laws in many African and Eastern Mediterranean countries prevented us from including more countries from these regions in this study.

## Conclusions

This study demonstrates that among countries that contribute the most to the global tobacco burden, there are still areas of nonalignment of tobacco laws with guidelines specified by article 11 of the FCTC. The gains made in global tobacco control in recent times can be consolidated by advocating for stronger tobacco regulations in compliance with the FCTC. Strong, effective, evidence-driven health warning labels are needed to protect and promote global public health.

## Abbreviations

FCTC: Framework convention on global tobacco control; WHO: World health organization; PDA: Principal display area.

## Competing interests

Both authors declare that they have no competing interests.

## Authors’ contributions

AA initiated the concept of the study, extracted and analyzed the data, and prepared the initial draft of the manuscript. JEC contributed to development of the methodology and the interpretation of results, and critically reviewed and revised the manuscript. Both authors read and approved the final manuscript.
